# Supplementary material for: Living donors kidney transplantation and oxidative stress: Nitric oxide as a predictive marker of graft function
Source: PLoS One. 2024 Sep 23;19(9):e0307824. doi: 10.1371/journal.pone.0307824 (PMC11419388; doi:10.1371/journal.pone.0307824)
Supplement: Attachment — Submitted filename: Response to reviewers IZEMRANE DJAMILA.docx [file pone.0307824.s002.docx]

Algiers, December 14th 2023.

Dear Academic Editors and Peer Reviewers

We thank you so much for the time you have devoted to reading and understanding this manuscript, as well as for your valuable comments and relevant remarks.

Further to your observations about the language of the article, I submitted the latter to Mr. Kamel Babouche, who holds a long experience in English-language teaching and training as a:

- Former high-school teacher of English, (1978-1990).
- Former English-language teacher trainer at a teaching school, (1990-1996).
- Former general inspector for English, (1996-2014).
- Former consultant at British Council Algeria, (2014-2018).

Mr. Babouche read through the article to see how best he could improve it in terms of language quality. Though he admitted there is much redundancy, his view is that the nature of the text, as well as the jargon in relation to the discipline allow for a certain amount of redundancy as regards the syntax. He reckons it is difficult to bring syntactic changes without running the risk of altering the meaning and comprehension of the article, and so limited himself to correcting a few grammatical and lexical mistakes. All mistakes are corrected in green in the manuscript titled "Revised Manuscript with Track Changes".

I also wish to draw your attention to the fact that English language -as a tool of teaching	and studying at university- has been introduced only recently in the Algerian universities and is gradually replacing French. This means that we have to make more effort to reach good standards of English and	we are doing our best for that purpose. Therefore I hope to rely on your understanding and indulgence	as regards the linguistic quality of the article.

In addition, we have responded to the 6 points required by PLOS ONE, as well as to the reviewers' comments.

PLOS ONE requirements

1- The formatting of the unmarked version complies with the recommendations of the link <https://journals.plos.org/plosone/s/file?id=wjVg/PLOSOne_formatting_sample_main_body.pdf>

<https://journals.plos.org/plosone/s/file?id=ba62/PLOSOne_formatting_sample_title_authors_affiliations.pdf>

2- Response to Emily Chenette, Editor-in-Chief of PLOS ONE, and Iain Hrynaszkiewicz, Director of Open Research Solutions at PLOS: We created the doi of our raw data according to the registrations on the https://plos.org/dryad-data/ website.

3- We have specified in the cover letter that funding from the two laboratories (Animal Biology and Physiology laboratory at the Ecole Normale Supérieure in Kouba, Algiers, Algeria and the Central Biology Laboratory at the CHU Lamine Debaghine, Bab El Oued, Algiers, Algeria) is used to finance our analyses.

4- We have mentioned in the cover letter and in green in the statistics section of the manuscript titled "Revised Manuscript with Track Changes" that our raw data are available to all scientific readers via <https://datadryad.org/stash/share/2amXBXayUq0oPVI2xg_IoF9_04VM3u7AFdfjfNtymFE> , lines 705 and 706.

5- The words "data not shown" have been removed from the text and appear in red in the "Results section/ Construction of prediction models of graft function" of the manuscript titled "Revised Manuscript with Track Changes", line 323.

6- We have included our full ethics statement in the "Methods" section of the manuscript titled "Revised Manuscript with Track Changes", lines 138,139 and 140.

Response to reviewer 1 Anonymous

1- As mentioned above, grammatical and lexical errors have been corrected by Mr. Kamel Babouche.

2- We would like to make a small clarification: we claim that the quantification of nitric oxide at day 6 in association of MDRD-eGFR D6, or MDRD-eGFR D21, or MDRD-eGFR M3 and/or donor age makes it possible to predict the glomerular filtration rate at 6 months, and consequently, the functioning of the kidney transplant in patients transplanted from living donors (see models 13, 14 and 15 table 6b) (Respectively, P= 0.000, r^2^ = 0.599, r^2^adj= 0.549 ; P= 0.000, r^2^= 0.548, r^2^adj = 0.497 ; P= 0.000, r^2^= 0.553, r^2^adj = 0.517). The other three biomarkers (malondialdehyde, myeloperoxidase and gluthathione s transferase) were not predictive in this study.

In many areas of the social and biological sciences, an r^2^ of about 0.50 or 0.60 is considered high (R.D. Cook and S. Weisberg (1999), Applied Regression Including Computing and Graphics, Wiley, p. 281), and the number of 10 observations per predictor is required, i.e. 30 observations for the 3 predictor variables (eGFR-MDRD D6 or D21 or M3, NO D6, age donor), so my sample of 32 patients is correct.

In addition, the value of the adjusted r^2^ differs slightly from that of the r^2^, which indicates the reliability of our models. The adjusted r^2^ shows the interest of the cumulative effect of adding a variable to a model. In multiple linear regression, the addition of a variable to a model increases systematically r^2^ without systematically increasing the value of the adjusted r^2^.

On another note, the P values of these three models are highly significant P=0.000 and the application conditions are met (normality of residuals, homoscedasticity of residuals and absence of multicollinearity). Also, the P values for the variable NO D6 were also significant in models 13, 14 and 15 (P= 0.002, P=0.011, P=0.000, respectively).

In addition, in model 13, which represents the best model, we can predict graft function at 6 months on the basis of quantification of creatininemia levels (allowing calculation of eGFR-MDRD) and nitric oxide levels in the blood on day 6. 1 unit of eGFR-MDRD D6 increases eGFR-MDRD M6 by 0.339 (coefficient of regression), similarly 1 unit of NO increases eGFR-MDRD M6 by 0.226 (coefficient of regression). The coefficients of the two serum biomarkers are relatively similar.

All these clarifications are provided from line 550 to line 565 and colored in green, in the document entitled "Revised Manuscript with Track Changes".

3- The first part of the results, from line 270 to line 310 and colored in red in the document entitled "Revised Manuscript with Track Changes" has been pruned to target the objectives. It is clear that some of the P values may be debatable.

4- The originality of this article is that, for the first time, modified MDRD equations have been developed to predict renal graft function at 6 months. These equations take into account two serum biomarkers nitric oxide (a biomarker associated with GFR according to references (36, 50, 51 and 52) and creatininemia, in addition to donor age. Several studies on donor parameters agree that donor age predicts graft function (references 55, 56, 57, 58 and 59).

However, this work deserves to be completed by validating these equations on a large cohort of kidney transplant recipients from living donors, or even testing these equations on a cadaveric cohort.

Response to reviewer 2 Mr. Seyed Reza Yahazadeh

1- Indeed, some readers found the manuscript long. Some parts of the text have been trimmed to make the results easier. All the parts concerned are marked in red (Results and discussion) in the document entitled "Revised Manuscript with Track Changes", from line 268 to line 310 and from line 496 to line 500. Also, two of the bibliographic references, namely 46 and 47, are no longer listed.

2- Some readers also found the tables difficult to read. A change has been made to the layout of tables 6a and 6b. The variables introduced and significant for each of the models are listed at the bottom of the table and not in the table itself. Table 7 has been completely modified to make the equations of the 15 models used to predict graft function at 6 months easier to read. These changes are marked in green in the document entitled "Revised Manuscript with Track Changes".

3- We have added a few details concerning surgery and donors as requested in the "Methods" section, namely ABO-compatible transplantation, spouses as unrelated living donors, uretero-vesical anastomosis in most cases, Celcior preservation fluid used and left kidney transplant, lines 135, 140 and 141. These details are marked in green in the document named "Revised Manuscript with Track Changes".

Sincerely yours,

Djamila IZEMRANE, Ali BENZIANE and Ahcène BAZ on behalf of the authors.

Corresponding author: Djamila IZEMRANE.

- Laboratory of Biology and Animal Physiology, Higher Normal School, Kouba, Algiers, Algeria, 16308.
- National Higher Veterinary School, Issad Abbes, Oued Smar, Algiers, Algeria, 16059.

Email: [d.izemrane@ensv.dz](mailto:d.izemrane@ensv.dz). [izemranedjamila@gmail.com](mailto:izemranedjamila@gmail.com). Phone number: +213 669 20 82 01 / +213 772 21 67 32.
