# Supplementary material for: Living donors kidney transplantation and oxidative stress: Nitric oxide as a predictive marker of graft function
Source: PLoS One. 2024 Sep 23;19(9):e0307824. doi: 10.1371/journal.pone.0307824 (PMC11419388; doi:10.1371/journal.pone.0307824)
Supplement: Attachment — Submitted filename: Response to reviewers IZEMRANE 9-5-24 PLOS ONE.docx [file pone.0307824.s003.docx]

Algiers, May 9th 2024.

Dear Academic Editor and Peer Reviewers

We thank you so much for the time you have devoted to reading and understanding this manuscript. We would also like to express our gratitude for the interest you have shown in our work, which is reflected in your pertinent comments and recommendations for improving the manuscript.

We have responded to the 2 points required by PLOS ONE, as well as to the reviewers' comments.

PLOS ONE requirements

1- Please note that the content of our financial disclosure was changed in the cover letter. We have removed the reference to personal contributions by Djamila IZEMRANE and Nacim HAMDIS.

2- As far as depositing our laboratory protocols in protocols.io is concerned, we feel that there is no need to make an additional contribution, given that these protocols are referenced (see methods section) and that the analysis methods are common methods with few modifications.

Response to reviewers

Reviewer#3

Despite these encouraging results, there is a need for evaluation and validation in a larger cohort and by other centers. This is mentioned in the lines 474, 475 of the document entitled "Revised Manuscript with Track Changes".

Cohorts for the creation and validation of predictive models are generated using the 20-80 or 30-70 method. Given that the number of patients transplanted to date at the University Hospital of Beb El Oued, with a follow-up of one year after the last patient in our cohort, is 58 patients. We cannot therefore validate a posteriori our predictive model of graft function at one year.

In addition, it is impossible to obtain D6 blood samples from these patients in order to repeat the NO assays for validation of the models predicting graft function at six months.

Reviewer#4

1- We have followed your recommendations by deleting some paragraphs from the introduction (lines 69-73, lines 79-83, lines 99-101 and lines 103-106) and from the discussion (lines 386,387, lines 389-395 and lines 400-407). All of these paragraphs are highlighted in red in the document entitled "Revised Manuscript with Track Changes". This has resulted in the deletion of references (10, 11, 22, 41, 42 and 43) and a change in numbering from 12th reference.

2- For patients with DGF compared to patients with prompt graft function, the activity of sMPO was higher for patients with DGF. The significant differences were observed at D6 and D8 (respectively, 112.04±44.32 vs. 49.89±39.49 U/L, *P=*0.023 and 126.81±59.29 vs. 47.48±30.72 U/L, *P=*0.01).

In addition, levels of sNO at D8 posttransplantation (99.86±44.22 vs. 45.77±40.0 µmole/L, *P*=0.027), higher in favour of the DGF group.

Reviewer#5

1- Typos and grammatical errors in the docuement entitled "Revised Manuscript with Track Changes" have been corrected : Line 59: recognized; Line 87: oxygen; Line 106: hemodialysis; Line 111: energy demanding, mitochondria; Line 118: In 2014, a; Line 120: measured; Line 124: living donors. It also; Line 125: transplantation as well; Line 130: October 2017 and November; Line 132: living donors.; Line 134: study. All; Line 135: The left kidney was transplanted and the type; Line 170: A volume of 100 ul, 2900 ul; Line 223: therapy consisting of calcineurin; Line 227: Table 1; Line 434 correlation results, which; Line 435: at 6M, shows; Line 502: aging; Line 577: race-free; Table 1: Rejection, Brother, Cousins; Table 5: Rejection. All these corrections are hilighted in green in the document entiteled "Revised Manuscript with Track Changes"

2- The data format has been corrected and hilighted in green, in tables 1 and 2, as well as in the paragraphs mentioning the t-test results, from line 246 to line 251 and from line 256 to line 259 in the document entitled "Revised Manuscript with Track Changes"
3- We have clarified the immunosuppressive therapeutic strategy of our transplant centre in the method section of the manuscript entitled "Revised Manuscript with Track Changes", from line 225 to line 232

4- The results of the bivariate correlations between mean and individual NO values and mean and individual calcineurin inhibitor values did not reveal any significance, on any of the follow-up days. The same was true for the correlations between mean and individual glomerular filtration rate values and mean and individual calcineurin inhibitor values. Similarly, we mentioned in Table 5 that there is no correlation between glomerular filtration rate and the choice of calcineurin inhibitor.

5- The MDRD equation used is for patients of non-African origin, see table 1.

6- The authors prefer to keep table 3, which expresses the values of the Pearson and Spearman coefficients "r" and "ρ", rather than the scatterplot, which shows the shape or trend of the correlation, especially for a small sample size with a wide dispersion of values in relation to the mean. The significant values of these correlations are highlighted in grey in table 3.

Sincerely yours,

Djamila IZEMRANE, Ali BENZIANE and Ahcène BAZ on behalf of the authors.

Corresponding author: Djamila IZEMRANE.

- Laboratory of Biology and Animal Physiology, Higher Normal School, Kouba, Algiers, Algeria, 16308.
- National Higher Veterinary School, Issad Abbes, Oued Smar, Algiers, Algeria, 16059.

Email: [d.izemrane@ensv.dz](mailto:d.izemrane@ensv.dz). [izemranedjamila@gmail.com](mailto:izemranedjamila@gmail.com). Phone number: +213 669 20 82 01
