# Supplementary material for: Living donors kidney transplantation and oxidative stress: Nitric oxide as a predictive marker of graft function
Source: PLoS One. 2024 Sep 23;19(9):e0307824. doi: 10.1371/journal.pone.0307824 (PMC11419388; doi:10.1371/journal.pone.0307824)
Supplement: Attachment — Submitted filename: Response to reviewers IZEMRANE 7-7-24 PLOS ONE.docx [file pone.0307824.s004.docx]

Algiers, July 7th 2024.

Dear Academic Editor and Peer Reviewers,

The authors would like to thank you for the time taking to reread and improve this manuscript.

We have responded to the three requirements of PLOS ONE and to the requests for corrections from Reviewer n#5.

PLOS ONE requirements

1- Please note that the content of our financial disclosure was changed in the cover letter (May 9^th^, 2024). We have removed the reference to personal contributions by Djamila IZEMRANE and Nacim HAMDIS.

2- As far as depositing our laboratory protocols in protocols.io is concerned, we feel that there is no need to make an additional contribution, given that these protocols are referenced (see methods section) and that the analysis methods are common methods with few modifications.

3- We have checked our original list of 56 references in the "Retraction Watch Database" Version 1.0.8.0. No references have been retracted. The final number of references is correct and in accordance with the text.

Response to reviewers

Reviewer#3

The authors thank you for your comment.

Reviewer#5

The authors thank you for your scrupulous examination of language, words and grammar, as well as for all your recommendations.

1- Typos and grammatical errors in the docuement entitled "Revised Manuscript with Track Changes" have been corrected and hilighted in green or in red :

line 62- kidney size (we have removed renal), line 85- endothelial cell dysfunction (we have added cell), line 112- we have removed "and ;" and "to", line 113- post-transplantation, line 130- blood samples were, line 142- using the thiobarbituric, we have removed "_" before An, lines 143, 150, 167, 168, 170, and 171 - we have respectively changed 375 µl, 25 µl, 100 µl, 3000 µl, 100 µl and 1 ml to 375 µL, 25 µL, 100 µL, 3000 µL, 100 µL and 1 mL, line 188- the Student's test, line 196- we have removed "throughout the first year", line 201- A p-value of < 0.05, line 207- we have removed "have been detransplanted" and we have put "had grafts removed", line 212- prednisone, line 214- Cyclosporine, line 244 and line 246- respectively were, line 248- we have removed "respectively", line 258- we have removed "a simplified equation for glomerular filtration rate estimated by modification of diet in renal disease", line 259- measure (typo), lines 271-298- In these tables all numbers are presented as "0.0" as indicated in the text, line 315- (Table 6a models), line 316- were tested, line 318- in Table 6 (Table 6b), line 321- we have removed "No variable is transformed.", line 328- Table 6a, line 343- Table 6b, line 344-349- we have removed MDRD-pGFR from the legend, line 354- (Table 4), line 355- Table 8, line 367- (1Y) post, lines 368 and 369- we have removed "A simplified equation for glomerular filtration rate estimated by modification of diet in renal disease", line 375- significantly, line 379- Our results are similar to previous studies, line 384- change to "donors, which were considered as controls, and recipients", line 393- analyzed, line 396- peripheral tissues, indicated , line 397- arginine , line 399- in arginine metabolism, line 400- C-reactive protein, line 407- "male and female patients who have had complications and those who have not.", line 418- results, which , line 420- r=0.451, (ρ=0.416 and r=0.451 respectively), line 422- (r_D2_=0.528, r_D3_=0.451, r_D6_=0.478, ρ_D8_=0.531, r_M1_=0.563, ρ_M3_=0,649 respectively), line 427- (P=0.017, ρ=0.431 and P=0.010, ρ=-0.450 respectively), line 429-432- change to "out. They were models 13, 14 and 15 (Table 6b: P= 0.000, r^2^ = 0.599, r^2^adj= 0.549; P= 0.000, r^2^ 455 =0.548, r^2^adj = 0.497; P= 0.000, r^2^= 0.553, r^2^adj = 0.517 respectively)." , line 433- we have added : "To put these findings in perspective, in many areas", line 440-we have removed "On another note," The p-values, line 442- Also, the p-values for, line 444- In addition, model 13, which, lines 446 and 447- One unit, line 452- In 2014, a study, line 455- measured (typo) GFR at day (typo) 5, lines 457 and 458- indicator of DGF and plasma levels of free thiols at 30 minutes and 90 minutes post-transplantation, line 464- blood flow and GFR relatively, lines 472 and 473- an increased asymmetric dimethylarginine (ADMA), line 476- endogenous NO inhibitors, ADMA and , line 477- as well as the NO precursors (arginine, citrulline and ornithine) with , line 478- were studied, line 482- 25 healthy subjects, NO showed, line 493- decreases NO production, line 494- of renal allografts revealed a significant increase of serum nitrate and episodes of acute rejection , lines 501 and 502- we have removed "The results of the most important of these are reported below." lines 508-510- we have removed the spaces before and after "/", line 511- we have removed "estimated glomerular filtration rate", line 518- donor's eGFR, line 526- donor eGFR, line 527-529- we have removed "This is a simple but useful guide for estimating graft function one year after renal transplantation, particularly in marginal donors (elderly patients, patients with hypertension and atherosclerotic disease), in the clinical setting", line 536- recipient GFR estimated at six months, line 537- Early prediction of GFR one year, line 538- transplantation was carried out in a large , line 540- period were analyzed (typo), line 549- variables, lines 552 and 553- trends towards measured GFR, line 553- no significant differences were observed, line 559- validated in multiple large international, line 561- the race-free 2021 CKD-EPI equation developed in individuals with native kidneys, lines 562 and 563- The P30 values (P30 being the proportion of eGFR within 30% of measured GFR) ranged from 73.0% to 91.3%, line 566- clinical, induction (typo)/primary immunosupressive (typo) treatment, lines 568 and 569- warm ischaemia and MDRD-eGFR at six months.

2- On line 469, there is no difference between NADPH and molecular NADPH. We have deleted "and molecular NADPH" from the sentence. We made a mistake in paraphrasing the authors of reference 43.

3- The final number of references is correct (56) and in line with the text.

Sincerely yours,

Djamila IZEMRANE, Ali BENZIANE and Ahcène BAZ on behalf of the authors.

Corresponding author: Djamila IZEMRANE.

- Laboratory of Biology and Animal Physiology, Higher Normal School, Kouba, Algiers, Algeria, 16308.
- National Higher Veterinary School, Issad Abbes, Oued Smar, Algiers, Algeria, 16059.

Email: [d.izemrane@ensv.dz](mailto:d.izemrane@ensv.dz). [izemranedjamila@gmail.com](mailto:izemranedjamila@gmail.com). Phone number: +213 669 20 82 01
